# Medication-induced Uveitis: An Update

**DOI:** 10.18502/jovr.v16i1.8254

**Published:** 2021-01-20

**Authors:** Kashif M Iqbal, Madeline W Hay, Parisa Emami-Naeini

**Affiliations:** ^1^(KM Iqbal and MW Hay contributed equally to this paper.); ^2^University of California Riverside School of Medicine, Riverside, CA; ^3^Western University of Health Sciences, Pomona, CA; ^4^Department of Ophthalmology and Vision Science, University of California, Davis, Sacramento, CA, USA

**Keywords:** Uveitis, Medication, Medication-induced Uveitis

## Abstract

Drug-induced uveitis is an uncommon but important cause of ocular inflammation. Uveitis can be seen in association with various systemic, topical, and intraocular medications. In this article, we review common medications associated with uveitis. Most cases of drug-induced uveitis resolve with termination of the suspected medication with or without administration of topical or systemic steroids. It is important for clinicians to readily identify medications that may cause uveitis in order to provide rapid treatment, avoid consequences of longstanding inflammation, and prevent costly and excessive laboratory testing.

##  INTRODUCTION

Uveitis is generally defined as inflammation in the uveal tract, which is composed of the iris, ciliary body, and choroid. Uveitis most commonly affects young, working-age adults, and it has been reported to be responsible for 5–20% of all cases of blindness in the United States and worldwide.^[1,2]^ According to the International Uveitis Study Group, uveitis is classified based on anatomic location of involvement, and can manifest as anterior, intermediate, posterior, and panuveitis.^[3,4,5]^ It can also be classified based on etiology, including infectious, non-infectious, and masquerade syndromes.^[3]^

Medications are a rare cause of uveitis, comprising <0.5% of cases.^[1,6]^ Drug-induced uveitis, although uncommon, can sometimes cause severe inflammation and is easily misdiagnosed. Hence, a high degree of suspicion is required to establish the diagnosis. Several criteria have been proposed to describe the causality of adverse events from medications, including a reaction that is frequently described and documented, recovery upon drug withdrawal, more severe reaction with higher doses, and recurrence with drug rechallenge; rarely does a drug meet all of these criteria.^[7]^ The pathogenesis of drug-induced uveitis is not fully understood, but various mechanisms have been proposed including a direct effect from topical application or intracameral injection, metabolite effects from drug detoxification, type III hypersensitivity reaction with immune complex deposition of antidrug antibodies, and antigens liberated from drug-induced death of microorganisms.^[8]^ Medications may also be broken down into free radicals that bind melanin in the uveal tract, which can cause toxicity and reduce melanin's ability to scavenge other free radicals, causing uveitis.^[8,9]^

In the current article, we review common systemic, topical, intracameral, and intravitreal medications associated with uveitis. New medications linked to uveitis that have been reported in the literature will also be highlighted.

##  TOPICAL MEDICATIONS

### Brimonidine

Brimonidine is an alpha-2 adrenergic agonist that is administered topically to reduce intraocular pressure. Acute granulomatous or non-granulomatous anterior uveitis with an elevated intraocular pressure has been reported with brimonidine use.^[10,11]^ The mechanism by which this inflammatory response occurs is largely unknown, but there is a higher risk in patients with history of allergic conjunctivitis from brimonidine use and in patients using drops for >12 months.^[12]^ Stopping the medicine usually resolves the inflammation and rechallenge results in recurrence of uveitis.^[11]^

### Prostaglandin analogues

Topical prostaglandin analogs increase uveoscleral outflow of aqueous humor, and are used in the treatment of glaucoma.^[13]^ Latanoprost is associated with a 5% risk of anterior uveitis within the first several months of treatment.^[14,15]^ A significant increase in anterior chamber cell and flare has been reported at three and six months after the initiation of latanoprost, travoprost, and bimatoprost.^[16]^ This may be due to breakdown of the blood–aqueous barrier and subsequent elevation of cytokines in the anterior chamber.^[16,17]^ Use of these drops has also been associated with the development of cystoid macular edema.^[15]^

##  INTRAOCULAR INJECTIONS

### Vancomycin

Intracameral vancomycin is used for prevention of endophthalmitis following cataract surgery.^[18]^ However, vancomycin use has been associated with hemorrhagic occlusive retinal vasculitis (HORV), often presenting with anterior chamber and vitreous inflammation as well as painless vision loss.^[18]^ All reported cases of HORV presented within 1–21 days (mean ∼8 days) after vancomycin use. These patients received vancomycin via intracameral injection, intravitreal injection, or through the irrigation bottle. Retinal vasculitis in most of these patients resulted in poor visual outcomes.^[18]^ The proposed mechanism by which this reaction occurs is via a delayed immune response to the drug itself.^[18]^

### Anti-vascular endothelial growth factor (anti-VEGF) agents

Anti-vascular endothelial growth factors (anti-VEGFs) such as bevacizumab (AvastinⓇ), ranibizumab (LucentisⓇ), and aflibercept (EyleaⓇ) are commonly used in the treatment of neovascular age-related macular degeneration, macular edema secondary to diabetic retinopathy and vascular occlusion, and proliferative retinopathies. After two years of anti-VEGF therapy, there is a two-fold increase in the prevalence of uveitis compared to disease-matched controls.^[19]^

Intraocular inflammation has been the dose-limiting variable for intravitreal use of ranibizumab.^[20]^ During the FOCUS trial, a 12% rate of uveitis was found following ranibizumab injection; however, the majority of these cases occurred prior to switching from the lyophilized formulation (no longer in use) to the liquid formulation, as well as prolonging the interval between injection and verteporfin photodynamic therapy.^[21]^ These changes were made to the protocol due to concerns that these factors increased the risk of uveitis.^[21]^ ANCHOR and MARINA clinical trials estimated that approximately 2% of patients receiving intravitreal injections of ranibizumab developed significant inflammation (classified as 3+ or more cell in the anterior chamber) within three weeks of injection.^[21,22]^ The HORIZON extension study evaluated the long-term safety of ranibizumab in patients who had completed the ANCHOR, MARINA, or FOCUS trials, and found that significant intraocular inflammation presented in 1.7–2.6% of the eyes receiving ranibizumab for one to three years.^[23]^ Another study reported that both bevacizumab and aflibercept were associated with a <1% risk of significant intraocular inflammation.^[24]^

The newest drug in the anti-VEGF family is Brolucizumab (BeovuⓇ), which comprises a humanized single-chain antibody fragment with a molecular weight of 26kDa, and recently received FDA approval for use in patients with wet age-related macular degeneration.^[25]^ The efficacy and safety of brolucizumab was evaluated and compared with aflibercept in the HAWK and HARRIER phase-three multicenter randomized trials, which found that uveitis was present in 2.2% and 0% of patients taking brolucizumab and aflibercept, respectively.^[25]^ About 90% of these cases were mild to moderate, and were treated successfully with topical corticosteroids.^[25]^ In the post-hoc analysis of the HAWK and HARRIER data, Mones et al^[26]^ reported that the incidence of intraocular inflammation was 4.6% in eyes treated with brolucizumab; 3.3% of patients developed retinal vasculitis with occlusive vasculitis in 2.1% of the eyes.^[26]^ In addition, 0.7% of the cases experienced at least moderate vision loss (≥15 ETDRS letters), and most of these events occurred in the first six months of drug use. In the same study, the incidence of intraocular inflammation in aflibercept-treated eyes was 1.1%, with at least moderate vision loss in 0.14%.^[26]^ The mechanism for intraocular inflammation secondary to anti-VEGF injections is not fully understood, but some experts suggest that it is due to the formation of anti-drug antibodies and subsequent hypersensitivity reactions to the medicine.^[27]^

### Triamcinolone acetonide

Intravitreal injection of triamcinolone acetonide, used in the treatment of non-infectious uveitis and macular edema, has been associated with sterile inflammation and non-infectious endophthalmitis. The reported incidence is between 0.5% and 9.7% of injections, and significantly increases with the use of preservatives.^[14]^

##  SYSTEMIC MEDICATIONS

### Cidofovir

Cidofovir is a nucleotide analog that inhibits viral DNA polymerase and is used for the treatment of infection with herpesviruses such as cytomegalovirus (CMV).^[28]^ Uveitis has been reported in 25–50% of patients after a median of 11 weekly doses of intravenous cidofovir.^[29,30,31,32]^ Uveitis is more common after intravitreal use of cidofovir.^[31]^ HIV patients who receive cidofovir for CMV retinitis are at higher risk of uveitis. In these patients, treatment with highly active anti-retroviral therapy (HAART) is an independent risk factor, likely secondary to higher circulating levels of cidofovir in setting of HAART.^[33]^ Moreover, it has been suggested that an elevated level of CD4+ T-cells in HIV+ patients is a risk factor for cidofovir uveitis, which makes it difficult to differentiate from immune recovery uveitis.^[33,34]^ Concurrent use of probenecid, on the other hand, can significantly decrease the rate of ocular side effects as it minimizes intraocular secretion of cidofovir.^[35]^ Cidofovir-induced hypotony is seen in 10–20% of HIV+ patients treated for CMV retinitis.^[31,32]^ The inflammation and hypotony usually respond to treatment with topical steroids and cycloplegic agents, but hypotony can persist for a long period of time.^[30,35,36]^

### Rifabutin

Rifabutin, used for prevention and treatment of Mycobacterium avium complex (MAC) in immunocompromised patients, can cause unilateral or bilateral anterior uveitis (usually associated with hypopyon), intermediate uveitis, posterior uveitis, or retinal vasculitis.^[14,37]^ Uveitis is usually dose-dependent and commonly occurs between two weeks and seven months following the initiation of therapy.^[38]^ Serum concentration and hence risk of inflammation increases with concurrent use of antifungal azoles, azithromycin, ethambutol, and some protease inhibitors through inhibition of hepatic cytochrome P450 enzymes.^[28,37]^ Notably, rifabutin-induced uveitis has also been reported in children and immunocompetent patients.^[28]^ Inflammation usually resolves with topical steroids.^[28]^

### Fluoroquinolones

Fluoroquinolones, which disrupt bacterial DNA synthesis by inhibiting DNA gyrase and DNA topoisomerase IV, have a broad spectrum of antibacterial activity indicated for the treatment of community-acquired pneumonia, sinusitis, chronic bronchitis, intra-abdominal abscesses, and skin infections.^[39,40]^ In Hinkle et al's retrospective analysis of 40 case reports of fluoroquinolone-induced uveitis, moxifloxacin was associated with 25 cases, but ofloxacin, ciprofloxacin, levofloxacin, norfloxacin, and gatifloxacin have also been reported to cause uveitis.^[1,28,40,41]^ The mean onset of uveitis is 13 days after the initiation of the drug (range: 0–20 days) and is usually bilateral; three characteristic findings in these patients include pigment dispersion with pigmented keratic precipitates and high intraocular pressure, diffuse iris transilluminating defects, and atonic pupils.^[40,41,42]^ Fluoroquinolone-induced uveitis is more common in women and has been associated with HLA-B27 and HLA-B51 haplotypes, suggesting a possible autoimmune predisposition.^[40]^ Uveitis is treated by discontinuing the drug and administration of topical corticosteroids.^[41]^

### Bisphosphonates

Bisphosphonates, pyrophosphate analogs that inhibit osteoclast activity, are commonly used to inhibit bone resorption in osteoporosis and metastasis to bone.^[28]^ They are strongly associated with anterior uveitis, scleritis, and episcleritis, with onset as early as 6 hr after intravenous administration.^[1,43,44]^ In a large retrospective pharmacovigilance study, zoledronate caused 51% of bisphosphonate-induced uveitis, with alendronate and pamidronate causing 23% and 13%, respectively.^[1]^ The bisphosphonates promote the release of inflammatory cytokines such as interleukin 1, interleukin 6, and tumor necrosis factor (TNF)-α, which can target the uveal tract.^[45]^ Resolution typically requires topical steroids and discontinuation of the medicine.^[28]^

### TNF-α Inhibitors

**Figure 1:**
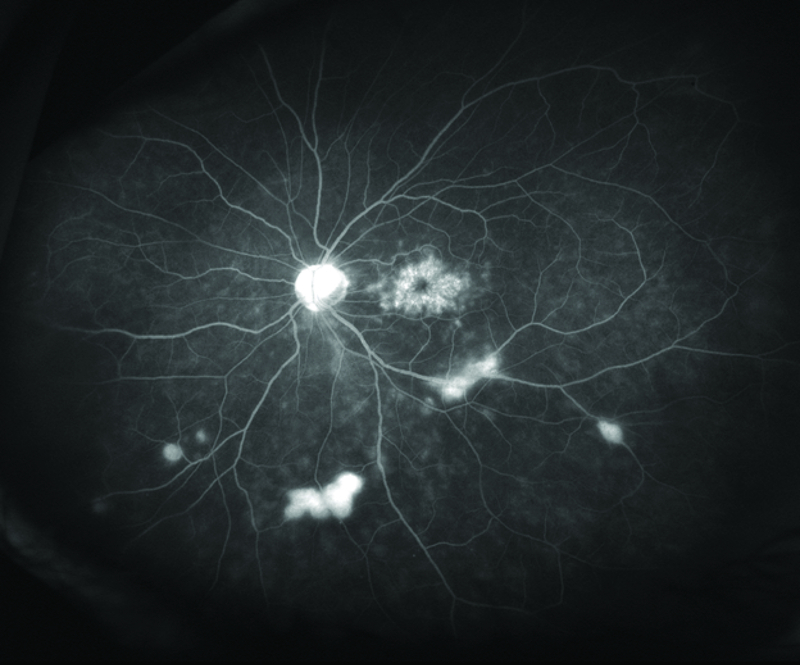
Fluorescein angiography of a 67-year-old female with history of rheumatoid arthritis treated with etanercept who developed uveitis and retinal vasculitis three months after the initiation of etanercept. Etanercept was discontinued and infliximab was started which resulted in resolution of ocular inflammation.

TNF-α inhibitors are a group of anti-inflammatory biologics that are used for the treatment of rheumatologic diseases such as rheumatoid arthritis, psoriasis, and inflammatory bowel disease, as well as scleritis and uveitis.^[41]^ Five anti-TNF- α drugs are currently approved for the use in autoimmune diseases, including four monoclonal antibodies (infliximab, adalimumab, golimumab, and certolizumab) and a soluble receptor blocker (etanercept).^[46]^ All of these medications have been paradoxically associated with the development of anterior uveitis and chorioretinitis. Inflammation is more common with etanercept but has also been reported with infliximab and adalimumab.^[47]^ The onset of uveitis is usually three weeks to six years after starting the therapy.^[41,47,48]^ Sarcoidosis has also been reported in patients using etanercept.^[46]^ The etiology of anti-TNF-α-induced uveitis is not fully understood, but it is hypothesized that decreased TNF-α levels leads to higher interferon levels and cytokine imbalances, resulting in auto-antibody formation and increased inflammation.^[49]^ Treatment involves discontinuation of the drug, with severe cases requiring systemic steroids^[41]^ [Figure 1].

**Figure 2:**
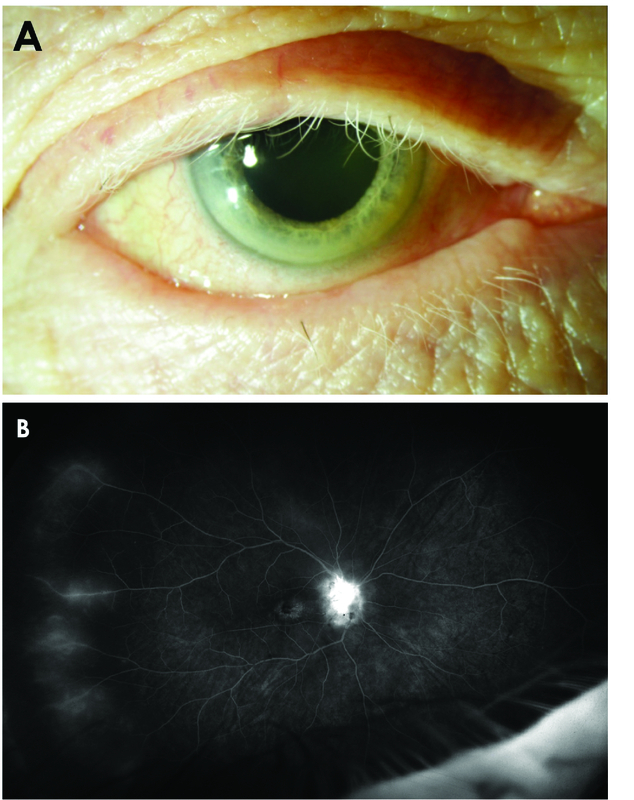
(A) 77-year-old man with history of malignant skin melanoma treated with nivolumab presented with blurry vision in both eyes. External exam showed poliosis of the eyelashes. Ultrawide field fluorescein angiography showed optic nerve and vascular leakage (B) which improved after intravitreal injection of triamcinolone acetonide.

**Figure 3:**
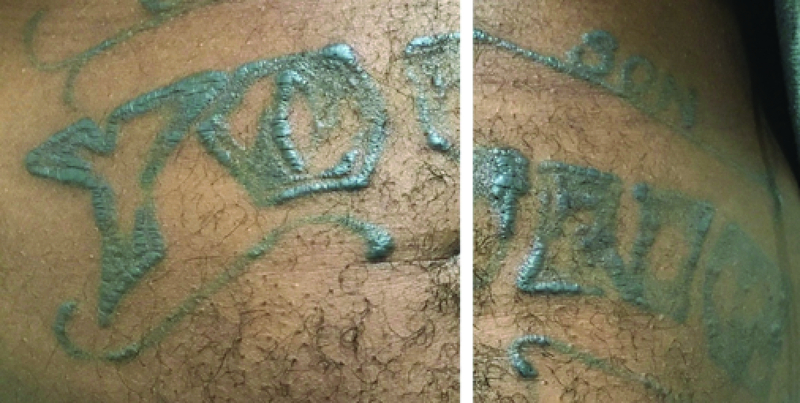
Inflamed, indurated skin tattoos in a young patient with tattoo uveitis.

### Immune Checkpoint Inhibitors 

Immune Checkpoint Inhibitors (ICIs) are emerging cancer immunotherapies used in metastatic melanoma and solid tumors.^[1]^ They upregulate the immune system by blocking immune checkpoints that are regulators of immune system, thus leading to activation of T-cells and an immune response to tumor cells.^[50,51]^ The different types of ICIs approved for use in cancer patients include a CTLA-4 inhibitor (ipilimumab), programmed cell death protein 1 (PD-1) inhibitors (pembrolizumab, nivolumab, and cemiplimab), and PD-1 ligand inhibitors (atezolizumab, avelumab, and durvalumab).^[14,52]^ These medications have recently been linked to ocular inflammation. Uveitis is seen in 1% of patients and is usually bilateral with onset between one and six months after the initiation of treatment.^[14,51,53,54]^ There are also reports of Vogt-Koyanagi-Harada (VKH) syndrome in patients receiving ICIs.^[1]^ Ocular inflammation in these patients is managed with topical or periocular steroids, but severe cases require systemic steroids and discontinuation of ICIs^[51]^ [Figure 2].

### Protein Kinase Inhibitors

Dysregulations in mitogen-activated protein kinase (MAPK) signaling pathways and BRAF gene mutations, seen in 50% of skin melanoma patients, can cause cell proliferation and cancer formation.^[14]^ BRAF inhibitors, such as vemurafenib and dabrafenib, and mitogen-activated protein kinases (MEK) inhibitors, such as trametinib, are new drugs of interest in the treatment of metastatic cutaneous melanomas. These medications have recently been linked to ocular inflammation.^[1]^ Uveitis usually occurs between six weeks and eight months after the initiation of treatment, and can present as anterior, intermediate, posterior, or panuveitis; resolution typically involves topical steroids.^[14]^ There are also reports of drug-induced VKH syndrome linked to the combination treatment with dabrafenib–trametinib.^[1]^

##  MISCELLANEOUS

### Vaccines 

There are reports of uveitis in association with BCG, influenza, hepatitis B, varicella, and human papilloma virus vaccines.^[14]^ Most of these cases respond to topical steroid treatment or observation and permanent vision loss is rare.^[14]^

### Other medications

Sulfonamides, including antibiotics (most commonly trimethoprim-sulfamethoxazole), diuretics, and sulfonylureas, have been associated with bilateral anterior uveitis usually within a week of drug initiation.^[28,41,55]^ Topical metipranolol, a nonselective β-blocker used to treat glaucoma, has been linked to granulomatous anterior uveitis most commonly when used at a higher concentration of 0.6%.^[28,56]^ Onset ranged from 2 to 31 months, and strong dechallenge and rechallenge data have also been reported.^[41,56]^ Other medications that can rarely cause uveitis include podophyllum, capsaicin, betaxolol, oral contraceptives, diethylcarbamazine, corticosteroids, quinidine, topiramate, and tuberculin skin tests.^[6,8,14,28]^ Almost all of these cases resolved with cessation of the medication and initiation of topical steroids.^[6,14,28]^

### Tattoo ink

There are multiple reports of patients with simultaneous bilateral uveitis and elevated inflamed skin tattoos. Skin biopsies from the indurated tattoos in these patients revealed granulomatous inflammation surrounding tattoo pigments, and some patients developed non-caseating granulomas in the draining lymph nodes corresponding to the location of the tattoos.^[57]^ An association between systemic sarcoidosis and tattoo uveitis has been reported by some authors, but uveitis can be found with or without a sarcoidosis diagnosis.^[57,58]^ Inflammation is more commonly seen in association with black ink, and there are reports of resolution of uveitis after removal of the skin tattoos.^[58]^ The etiology has not been clearly defined, but a type IV delayed hypersensitivity reaction has been proposed^[58]^ [Figure 3].

##  CONCLUSION 

Drug-induced uveitis is seen in association with a growing list of various topical, intraocular, and systemic medications. Although uncommon, medication-induced uveitis can cause severe, vision-threatening inflammation, and will increase in frequency with the development of new medications. The diagnosis is often made by a thorough history and evaluation of medication list, and after ruling out other potential infectious or non-infectious etiologies of ocular inflammation. Early identification of uveitis and rapid treatment can lead to decreased morbidity and complications of longstanding uveal inflammation, thus improving visual outcomes. Most of these cases respond to cessation of the insulting agent in conjunction with topical and/or systemic corticosteroids.^[14]^

##  Financial Support and Sponsorship

None.

##  Conflicts of Interest 

There are no conflicts of interest.
